# Laser‐Triggered Small Interfering RNA Releasing Gold Nanoshells against Heat Shock Protein for Sensitized Photothermal Therapy

**DOI:** 10.1002/advs.201600327

**Published:** 2016-10-19

**Authors:** Zhaohui Wang, Siwen Li, Min Zhang, Yi Ma, Yuxi Liu, Weidong Gao, Jiaqi Zhang, Yueqing Gu

**Affiliations:** ^1^ State Key Laboratory of Natural MedicinesJiangsu Key Laboratory of Drug ScreeningDepartment of Biomedical EngineeringSchool of EngineeringChina Pharmaceutical UniversityNo. 24 Tongjia LaneGulou DistrictNanjing210009China

**Keywords:** gold nanoshells, heat shock proteins, photothermal therapy, siRNA delivery

## Abstract

The resistance of cancer cells to photothermal therapy is closely related to the overexpression of heat shock proteins (HSPs), which are abnormally upregulated when cells are under lethal stresses. Common strategies that use small molecule inhibitors against HSPs to enhance hyperthermia effect lack spatial and temporal control of drug release, leading to unavoidable systemic toxicity. Herein, a versatile photothermal platform is developed which is composed of a hollow gold nanoshell core densely packed with small interfering RNAs against heat shock protein 70 (Hsp70). Upon near infrared light irradiation, the small interfering RNAs can detach from gold surface specifically and escape from endosomes for Hsp70 silencing. Meanwhile, the temperature increases for hyperthermia therapy due to the high photothermal efficiency of the nanoshells. Efficient downregulation of Hsp70 after light activation is achieved in vitro and in vivo. Ultimately, the light‐controlled dual functional nanosystem, with the effects of Hsp70 silencing and temperature elevation, results in sensitized photothermal therapy in nude mice model under mild temperature. This strategy smartly combines the localized photothermal therapy with controlled Hsp70 silencing, and has great potential for clinical translation with a simple and easily controlled structure.

## 1 Introduction

Photothermal therapy (PTT) for tumor ablation has experienced explosive development in recent years beyond the orthodox treatment regimens like chemo‐ and radiotherapy.^1^ PTT requires energy absorbing agents delivered into tumors, which can subsequently transfer near infrared (NIR) laser energy into heat, elevate local temperature, and ablaze cancer cells specifically.^2^, ^3^, ^4^, ^5^, ^6^ Despite the advantages, heat treated cancer cells can rapidly acquire thermoresistance with abnormally elevated expression of heat shock proteins (Hsps), leading to insufficient apoptosis, increased cell viability, and tumor recurrence.^7^

Hsps, especially Hsp70s are omnipresent molecular chaperones which facilitate correct protein folding and are expressed more abundantly under hyperthermia.^8^ Hsp70 has also been evidenced to exert antiapoptosis effects by inhibiting caspase‐3 activation and blocking kinase pathways activated by cellular stresses.^9^, ^10^, ^11^, ^12^ These cytoprotective effects render tumor resistant to PTT induced apoptosis. Therefore, the inhibition of Hsp70, a recognized target in cancer treatment, may sensitize cancer cells to PTT. The combination of hyperthermia and Hsp70 inhibition has been studied in several reports^13^, ^14^, ^15^ using microwave or magnetic heating. In most of these attempts, the inhibition of Hsp70 was achieved through systemic injection of small molecule inhibitors. Although relatively enhanced tumor ablation efficacy could be obtained, these treatments still suffer from the limitations of conventional chemotherapy as systemic toxicity, which overshadows the shining points of PTT itself. Moreover, overdose of hyperthermia may cause serious side effects or damages to peripheral healthy tissues. As a result, it is highly desirable to develop a photothermal platform that can generate sufficient heat, inhibit Hsp70 with spatial and temporal control, and achieve satisfying tumor ablation with mild hyperthermia.

Gold nanoshell is a perfect candidate to form such a platform with excellent photothermal efficiency and controlled release of accommodated multiple theranostic modalities on‐demand.^16^, ^17^, ^18^ For deep tissue PTT, gold nanoshells are prevailing over conventional NIR dyes because of their greater efficiencies in cross‐section absorption and light–heat conversion within NIR range.^19^, ^20^ More importantly, the versatile surface chemistry of gold not only enables easy modification of chemotherapeutics or nucleotides on these materials, but also confers light‐controlled release of the therapeutic cargos at the same time.^21^, ^22^ One of the most actively pursued cargo is small interfering RNAs (siRNAs), as RNA interference has demonstrated great potential for directly controlling the flow of genetic information in cancer treatment.^23^, ^24^ Upon NIR light irradiation, the thiol–gold bond will be thermalized and disassociate, leading to the release of siRNA. At the same time, localized cavitation will be generated and physically breach endosome membrane for endosomal escape of siRNAs, thus avoiding enzymatic degradation.^25^, ^26^ In addition, to date, gold nanoshells are the only metal nanomaterials that have entered clinical trials for photothermal therapy.^27^

**Scheme 1:**
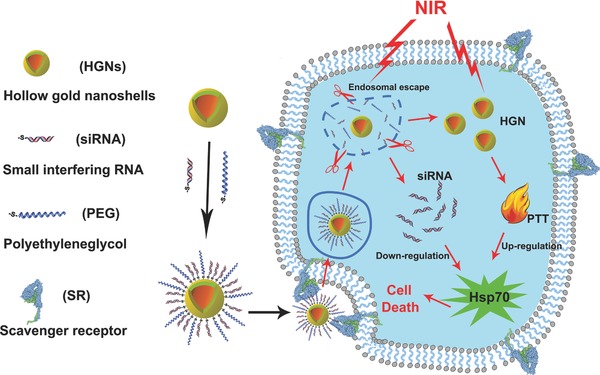
Schematic illustration of the nanoconstruct design to facilitate efficient siRNA delivery and sensitized photothermal therapy upon near infrared light irradiation.

In this study, a simple and versatile nanoplatform was established using hollow gold nanoshells conjugated with siRNAs against Hsp70 (**Scheme** **1**). This platform aimed to neutralize Hsp70 expression in tumor cells for enhanced tumor ablation with medium level hyperthermia and to avoid cell necrosis triggered by excessive heat, an undesirable type of cell death that may cause detrimental inflammatory and immunogenic responses. In the first step, we examined the stability of our nanoconstructs in environments containing different biological ingredients and light‐controlled siRNA release. Then, the cellular uptake and intracellular fate of the nanoconstructs were carefully studied. Thereafter, near infrared laser triggered gene silencing was confirmed. Finally, the therapeutic efficacy of PTT with the nanoconstructs was evaluated in vitro and in vivo.

## 2 Results and Discussion

### 2.1 Preparation and Characterization of the Nanoconstructs

**Figure 1:**
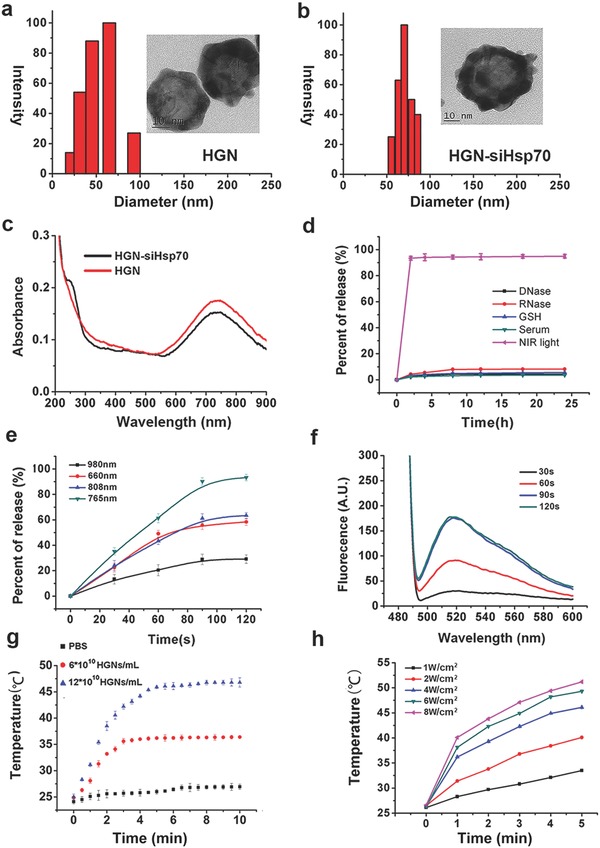
Preparation and characterization of hollow gold nanoshells (HGN) and HGN conjugated with small interfering RNAs (siRNAs). a) Dynamic light scattering (DLS) and transmission electron microscope (TEM) characterization of HGN. b) TEM and DLS data of HGN‐siHsp70 with PEG stabilization. c) UV–vis spectrum of HGN and HGN‐siRNA. d) Fluorescence spectra of the supernatants of HGN‐siHsp70 after different treatments. e) siRNA release from HGN‐siHsp70 under lasers of different wavelengths. f) Fluorescence spectra of the supernatants of HGN‐siHsp70 after laser treatment of different periods of time. g) Photothermal effects of HGN‐siHsp70 of different concentrations in solution. h) Temperature increases of HGN‐siHsp70 solutions under lasers of different power densities.

The hollow gold nanoshells (HGNs) were prepared through the modification of previously reported methods,^28^, ^29^ which showed an intense absorption peak at NIR region (Figure S1, Supporting Information). The size of HGN was ≈50 nm in diameter, as measured by dynamic light scattering (DLS) and transmission electron microscopy (TEM) (**Figure** **1**a). After siRNAs and polyethylene glycol (PEG) conjugation, the average hydrodynamic diameter of HGN obviously increased from 50 to about 70 nm (Figure 1b). The zeta potential of the negatively charged HGN was about −40 mV and decreased to nearly −60 mV when siRNAs were coated on particle surface, as siRNAs were also negatively charged (Figure S2, Supporting Information). The successful conjugation of siRNAs to HGN was further validated by ultraviolet–visible (UV–vis) spectrum (Figure 1c). After siRNA coating, a new absorption peak appeared at 260 nm, coinciding with the characteristic peak of RNA. Moreover, the connection of HGN and siRNAs by Au—S bond was proved by dithiothreitol (DTT) treatment, which could break Au—S bond through thiol‐exchange reaction and led to a drastic fluorescence increase of siRNAs in the supernatant (Figure S3, Supporting Information). PEG as an electrically neutral substance was used to prevent nanoparticle aggregation and reduce the immunogenicity of the nanoconstruct. The loading efficiency of siRNAs on HGN was then calculated as higher than 70%, and the HGN to siRNA ratio was calculated as 1:200 (Figure S4, Supporting Information).

### 2.2 Stability and Laser Triggered siRNA Release

For our nanoconstructs to be effective in biological environments, they must be stable against damage by intracellular enzymes and reductants. The stability test showed that nearly no siRNAs were detached from HGN treated with glutathione (GSH), serum, deoxyribonuclease (DNase), or ribonuclease (RNase). In contrast, NIR laser led to a substantial siRNA release of more than 90% (Figure 1d). These results verified the stability of this nanoconstruct and NIR light triggered siRNA release. The most appropriate laser wavelength to induce siRNA release was then determined. 765 nm laser resulted in the highest siRNA release rate, suggesting that the laser resonant with gold shell plasmon was the most efficient to break the Au—S bond (Figure 1e). To further probe the 765 nm light triggered siRNA release, solutions of the nanoconstructs were subjected to laser for different periods of time (Figure 1f). The fluorescence intensity of the supernatants gradually increased along with the extension of laser exposure time. 90 s irradiation resulted in the strongest signal, and longer irradiation of 120 s did not further increase the fluorescence. It was also found that only power densities higher than 4 W cm^−2^ could release more than 90% siRNAs within 90 s (Figure S5, Supporting Information). Then, the photothermal effect of the HGN conjugated with siRNAs against Hsp70 (HGN‐siHsp70) was evaluated (Figure 1g). The exposure of the solutions containing 6 × 10^10^ and 12 × 10^10^ HGNs per mL to 4 W cm^−2^ laser increased their temperatures to 37 and 48 °C, respectively, as compared to 27 °C of the phosphate buffered saline (PBS) control. It was also verified that larger power density could raise solution temperature more rapidly (Figure 1h). As a photothermal agent and carrier, the photothermal stability of hollow gold nanoshells was tested by examining the UV–vis spectrum and hydrodynamic diameter before and after laser irradiation (Figure S6, Supporting Information). The absorption peak of hollow gold nanoshells gradually shifted to shorter wavelengths as the irradiation time increased. After 4 min, the absorption peak drastically shifted from about 700 to 600 and 550 nm (Figure S6a, Supporting Information). Also, the hydrodynamic diameter obviously decreased from 50–60 to less than 30 nm after laser irradiation (Figure S6b,c, Supporting Information). Therefore, morphological changes may occur when the gold nanoshells were subjected to laser irradiation after a certain period of time. Even so, such changes would not influence the hollow gold nanoshells as a photothermal agent and carrier, as we have demonstrated the laser triggered release of siRNAs within 2 min (Figure 1f), and photothermal effect in solution (Figure 1g). These data indicated that our nanoconstructs could serve as an efficient photothermal coupling agent.

As 90 s laser irradiation at 4 W cm^−2^ could only slightly raise solution temperature, it was possible that the efficient siRNA release triggered by NIR light was not due to temperature increase. We then monitored the release of siRNA in the solutions thermalized by direct water bath and NIR laser, respectively (Figure S7, Supporting Information). No obvious siRNA release was observed in the thermal heating group as the temperature increased from 25 to 40 °C. In comparison, under NIR laser irradiation, the siRNAs rapidly detached from HGN at a temperature higher than 30 °C, with more than 80% released at 35 °C.This finding implied that a nonthermal mechanism may play a role in NIR light triggered siRNA release. One possible explanation is that during laser irradiation, gold nanoshells undergo a rapid local temperature increase, which is enough to break the Au—S bond but not sufficient to elevate the ambient solution temperature.^30^

### 2.3 Cellular Uptake and Intracellular Fate of the Nanoconstructs

**Figure 2:**
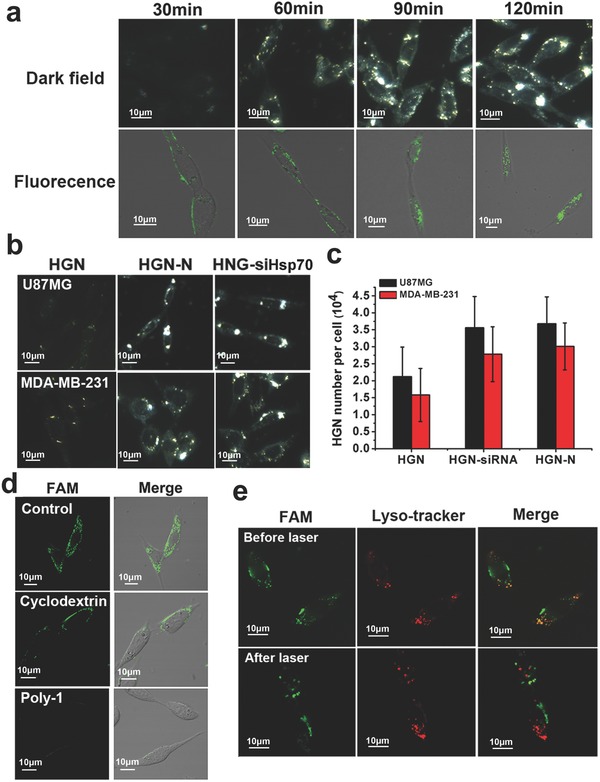
Uptake of HGN‐siHsp70 in U87MG cells intracellular fate of siRNAs after endocytosis. a) Cellular uptake of HGN‐siHsp70 within 120 min measured by dark field and laser confocal microscopy. b) Dark field images of U87MG and MDA‐MB‐231 cells after 120 min incubation with HGN, HGN conjugated with nonsense sequences (HGN‐N), and HGN‐siHsp70. c) Quantification and comparison of gold content in cells treated with HGN with and without oligonucleotide modification using ICP‐AES. d) Laser confocal images of the uptake of HGN‐siHsp70 in U87MG cells pretreated with different chemical inhibitors. e) Endosomal escape of HGN‐siHsp70 after laser treatment, red: lysosome, green: HGN‐siRNA, and yellow: colocalization of HGN‐siHsp70 and endolysosome.

The cellular uptake process of U87MG) cells to HGN‐siHsp70 was observed using hyperspectral microscopy and confocal laser scanning microscopy (**Figure** **2**a). The reflection signal gradually increased in the cytoplasm within the first 90 min incubation and remained stable afterward. As the siRNAs used in this study was modified with fluorescent dye Carboxyfluorescein (FAM), the intracellular uptake of HGN‐siHsp70 could also be visualized through laser confocal microscopy. Consistent with the reflection signal of dark‐field image, the intracellular fluorescence accumulation also peaked at 90 min and showed no obvious increase at 120 min. Therefore, 90 min was selected as the preferable incubation time for further experiments. The highly efficient cellular uptake of the nanoconstructs inspired us to explore the association of siRNA coating with endocytosis. To this end, U87MG and MDA‐MB‐231 cells were incubated with bare HGN or RNA coated HGN, and intracellular nanoparticle accumulation was then studied by hyperspectral microscopy and inductively coupled plasma atomic emission spectroscopy (ICP‐AES). After 90 min incubation, bare HGN only showed marginal reflection signal in U87MG and MDA‐MB‐231 cells, possibly due to insufficient cellular uptake. Contrastively, conjugated with siRNAs or nonsense RNA sequences (designated as HGN‐siHsp70 and HGN‐N, respectively, and HGN to sequence ratio being 1:200), HGN demonstrated an obvious increase of intracellular accumulation, as evidenced by the evidently brighter signal in both cell lines (Figure 2b). The quantification of nanoparticle number using ICP‐AES further confirmed our observation (Figure 2c). Of note, the surface modification of siRNA against Hsp70 and the nonsense sequence resulted in comparable numbers of HGN in cells, indicating that the increased cellular uptake was independent of the difference between oligonucleotide sequences. It was then speculated that the degree of cellular uptake was correlated with the loading density of siRNA. To verify this claim, HGN conjugated with different numbers of siRNA (50:1, 100:1, 200:1, 400:1, and 800:1) were incubated with U87MG cells for ICP‐AES quantification (Figure S8, Supporting Information). The endocytosis level of the nanoconstructs increased sharply from 50:1 to 100:1 and 200:1, but no drastic change occurred when the loading density was higher. Our group has previously reported a positive correlation between DNA number on gold nanoparticle surface and the detection signal of gold nanoparticle based molecular beacons, but did not investigate the influence on cellular uptake.^31^, ^32^ This finding may provide a more comprehensive insight into the endocytosis of oligonucleotide modified nanoarchitecture for diagnostic and therapeutic purposes.

Previous studies of Mirkin and co‐workers^33^, ^34^ have revealed that scavenger receptor on cell surface played an important role in cellular uptake of gold nanoparticles densely packed with DNA sequences. However, the endocytosis pathways of oligonucleotide‐coated gold nanoshells have not been studied yet. Our nanoconstructs with similar 3D morphological characteristics may also enter cells through scavenger receptor mediation. To verify this possibility, U87MG cells were pretreated with polyinosinic acid (Poly I), a well‐known ligand of scavenger receptor and then incubated with the nanoconstructs (Figure 2d). Poly I pretreatment resulted in almost complete fluorescence reduction, which was indicative of the major involvement of scavenger receptor in the cellular uptake of the nanoconstructs. We also tested the blocking effect of methyl‐β‐cyclodextrin, a pharmacological inhibitor of lipid‐rafts, on the endocytosis of the nanoparticles. Laser confocal imaging showed that pretreatment with methyl‐β‐cyclodextrin could potently reduce cellular association of HGN‐siHsp70. Therefore, the endocytosis of the nanoconstructs was most probably through scavenger receptor recognition and lipid‐rafts trafficking. We then investigated the intracellular fate of our nanoconstructs and the siRNAs delivered by them (Figure 2e). Before laser irradiation, a large number of particles were colocalized with endolysosomal compartments stained with LysoTracker Red. In comparison, 20 min after laser treatment, most of the green fluorescence was separated from the red, suggesting successful endosomal escape of siRNAs. Escape of the nanoparticles may be explained by the vapor bubble formation that ruptures the endosomes without damaging the siRNAs or the cells. The laser‐activated endosomal escape of siRNAs from gold nanoshells ensures efficient downregulation of target proteins.^34^

### 2.4 Laser Controlled Downregulation of Hsp70

**Figure 3:**
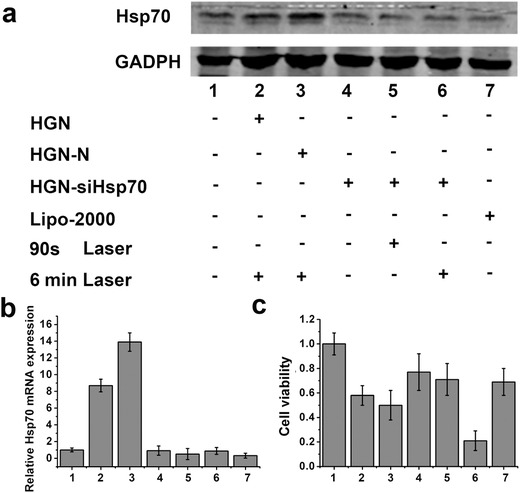
Gene silencing efficacy of HGN‐siHsp70 in different treatment conditions. a) Western blot assay of heat shock protein 70 (Hsp70) expression. b) Relative mRNA expression of Hsp70 determined by real time PCR. c) Viabilities of cells treated under the above conditions determined by MTT assay.

In hyperthermia treatment, tumor cells commonly overexpress Hsp70 to prevent heat damage. Herein, we tested the efficacy of our nanoconstructs to regulate Hsp70 expression in photothermal conditions. The downregulation of Hsp70 by the nanoconstructs was tested in U87MG cells. Real‐time polymerase chain reaction (RT‐PCR) and western blot assays were carried out to evaluate the knockdown of Hsp70 (**Figure** **3**a,b). In Figure 3a, “‐” indicated cells were not subjected to corresponding conditions, while “+” meant cells were treated under such conditions. Among these different treatments, 90 s laser irradiation was used to induce siRNA release in cells without temperature elevation, while 6 min laser could both release siRNAs and generate enough heat for photothermal therapy. As the control group, Group 1 showed the normal level of Hsp70 expression in cells. Because Hsp70 is a constitutive protein commonly expressed in cells, its expression is evident even without heat stimulation. Cells in Group 2 and Group 3 demonstrated increased Hsp70 expression compared with Group 1, due to the photothermal effect induced by HGN and 6 min laser irradiation. Of note, cells incubated with HGN‐N showed higher mRNA and protein levels of Hsp70, possibly as a result of improved cellular uptake of gold nanoshells by RNA coating, which generated more heat upon laser irradiation.

We then examined the gene silencing efficacy of HGN‐siHsp70 under different conditions. For comparison, Group 7 was set by incubating cells with siRNAs delivered by Lipofectamine2000 (Lipo), a recognized method for gene silencing. Cells treated with HGN‐siHsp70 but no laser irradiation (Group 4) showed no obvious downregulation of Hsp70 compared with the control group, possibly due to inefficient siRNA release without laser. For Groups 5 and 6, the expression of Hsp70 was evidently decreased compared with Group 1, which was comparable to Group 7. The efficient gene silencing efficacy of these two groups was most probably caused by laser triggered release of siRNAs and the subsequent endosomal escape. Of note, even subjected to the “heating” condition with 6 min laser irradiation, the expression of Hsp70 of Group 6 was also effectively downregulated to the level similar to Group 7. Therefore, HGN‐siHsp70 was potent to neutralize the expression of Hsp70 with comparable efficacy with Lipo. Also, effective gene silencing only occurred with NIR light activation, suggesting a precise gene silencing strategy without affecting unirradiated cells.

### 2.5 In Vitro Antitumor Efficacies of Different Treatments

The effective downregulation of Hsp70 prompted us to explore its effects on photothermal therapy. Seven groups of cells were subjected to the treatments as used above. 3‐(4,5‐Dimethylthialzol‐2‐yl)‐2,5‐diphenyltetrazoliumbromide (MTT) assays were then carried out after PTT (Figure 3c). PTT using bare HGN and HGN conjugated with nonsense sequence (HGN‐N) led to the losses of cell viability of 42.1% and 51.3%, respectively. The slightly increased inhibition effect of the latter was probably due to enhanced intracellular accumulation HGN. Silencing Hsp70 only without PTT did not cause obvious cell inhibition, either by the nanoconstructs or Lipo (Groups 5 and 7). Without laser activation, HGN‐siHsp70 treated cells exhibited a survival rate of 77%, implying the ineffective cell gene silencing in this group (Group 4) and the negligible toxicity of the “inactivated nanoconstructs.” As expected, the most significant decrease in cell viability was observed in Group 6 treated with both PTT and HGN‐siHsp70.

**Figure 4:**
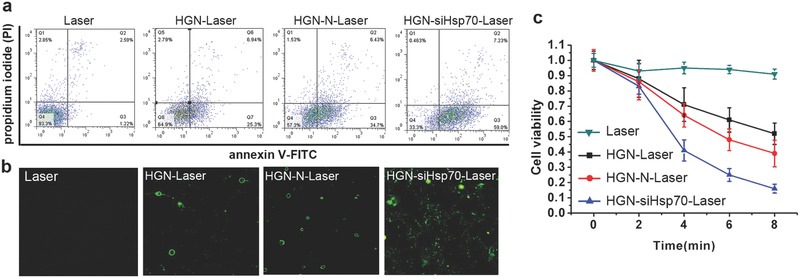
Determination of cell death pathways after photothermal therapies (PTT) with different nanoconstructs, where Laser representing control treatment with only 765 nm laser exposure, HGN‐Laser representing PTT in cells treated with bare HGN (only with PEG stabilization), HGN‐N‐Laser representing PTT in cells treated with HGN coated with nonsense sequences, and HGN‐siHsp70‐Laser representing PTT in cells treated with HGN coated with small interfering RNAs against Hsp70. Annexin V‐fluorescein isothiocyanate (annexin V‐FITC) and propidium iodide (PI) staining was used for apoptosis assay. a) Flow cytometry of the apoptosis of U87MG cells. b) Laser confocal microscopy examination of the apoptosis of U87MG cells after different PTT treatments. c) Cell viabilities of different PTT strategies within 8 min.

The in vitro tumor inhibition efficacy of PTT using our nanoconstructs was further examined with different laser exposure time from 0 to 8 min (**Figure** **4**c). The high cell inhibition ratio higher than 85% could be obtained upon PTT using HGN‐siHsp70 with 8 min laser treatment, which was unattainable by HGN and HGN‐N without siRNA delivery. Given the cellular uptake of HGN‐N and HGN‐siHsp70 was similar, the cell viability difference was mainly caused by Hsp70 downregulation of the latter, which sensitized tumor cells to heat. To date, most photothermal therapies reported require excessive heat to achieve a sufficient tumor inhibition through cell necrosis, which is undesirable as inflation and tumor metastasis may be induced by this abnormal type of cell death.^35^ As the temperature for PTT in this study was relatively moderate (lower than 48 °C), the cell death may be mainly induced by apoptosis.^36^ We examined the pathways of cell death using annexin V‐fluorescein isothiocyanate (annexin V‐FITC) and propidium iodide (PI) staining (Figure 4a,b). Flow cytometry showed that our PTT regimens mainly caused cell apoptosis rather than necrosis, with HGN‐siHsp70 leading to the largest number of apoptotic cells. Laser confocal imaging was consistent with flow cytometry results. Cells in all the treatment groups were stained with annexin V‐FITC on cell membrane but not with PI inside cells, indicating the apoptosis‐induced cell death. These results implied that the silencing of Hsp70 played the major role in enhancing PTT effect at apoptosis level.

### 2.6 Biodistribution and In Vivo Photothermal Efficiency

**Figure 5:**
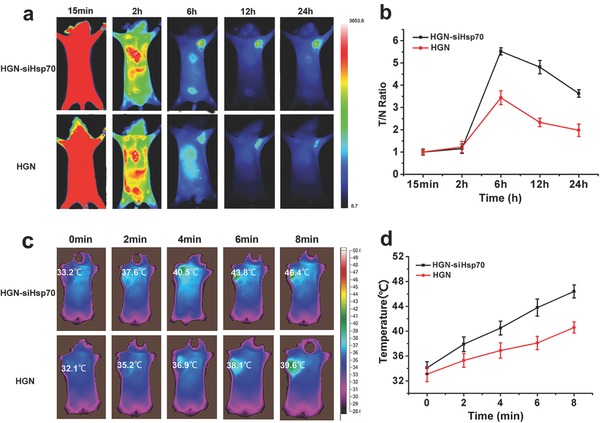
In vivo biodistribution and photothermal effects of HGN and HGN‐siHsp70. a) Fluorescence imaging of HGN‐siHsp70 and HGN at 15 min, 2 h, 6 h, 12 h, and 24 h after intravenous injection. b) Tumor to normal tissue ratios of U87MG tumor bearing mice at different time points after injection. c) Infrared thermal images of U87MG tumor bearing mice i.v. injected with HGN and HGN‐siHsp70 within 8 min under 765 nm laser irradiation. d) Temperature changes on tumor sites according to the imaging in (c).

After in vitro studies, we accessed whether the nanoconstructs could effectively accumulate in tumor after intravenous injection. For in vivo imaging, bare HGN and HGN‐siHsp70 were conjugated with the NIR dye ICG‐Der‐02 prepared in our lab to form HGN‐NIRdye and HGN‐siHsp70‐NIRdye, respectively. Tumor bearing mice were then imaged at different time points under the NIR fluorescent imaging system (**Figure** **5**a). Both HGN‐NIRdye and HGN‐siHsp70‐NIRdye showed obvious tumor retention at 6 h, which remained observable at 24 h post injection. This selective tumor accumulation was possibly caused by the enhanced permeation and retention effect. More importantly, compared with HGN‐NIRdye, mice treated with HGN‐siHsp70‐NIRdye showed more obvious tumor fluorescence at each time point. The tumor/normal tissue ratio (T/N ratio) was plotted against each time point, which was consistent with the in vivo imaging (Figure 5b). This observation was consistent with the cellular uptake experiments where surface coating of siRNAs increased cell association with gold nanoparticles. The effective tumor accumulation of the nanoconstructs promised effective in vivo photothermal efficacy. Then, the temperature changes at tumor sites after intravenous injection upon laser irradiation were monitored using a thermal camera (Figure 5c,d). We compared the temperature elevation in mice injected with HGN and HGN‐siHsp70 at different time points. Tumor temperatures in both groups increased along with irradiation time. Notably, the group with HGN‐siHsp70 injection showed more rapid temperature elevation compared with that of the HGN treated group. After 8 min laser exposure, the tumor temperature of HGN‐siHsp70 group reached 46.4 °C in comparison with HGN group of 39.6 °C, which was sufficient for in vivo tumor ablation. This observation coincided with the in vivo distribution results, pointing to the benefit of siRNA modification to enhance tumor retention of nanoparticles for improved photothermal effect.

### 2.7 In Vivo Photothermal Therapy

**Figure 6:**
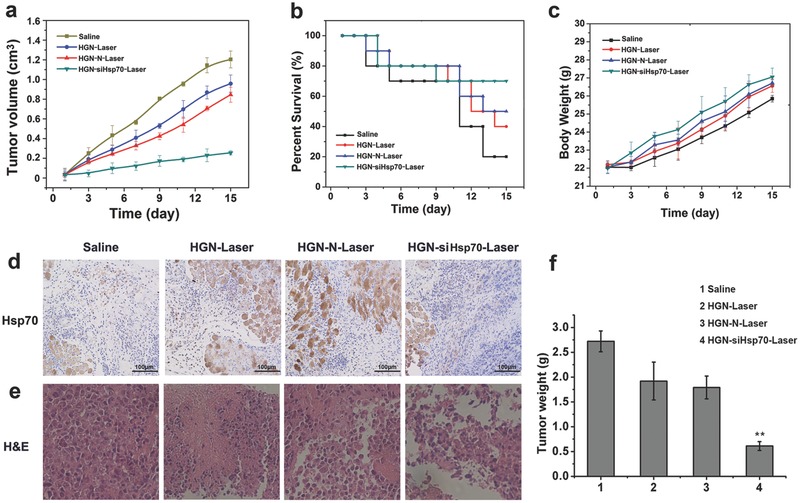
Photothermal therapy of tumor bearing mice treated with different nanoconstructs. a) Tumor growth curves of mice during the 15 d treatment. b) Survival rates of mice of different treatment groups. c) Animal weight changes of different groups during the treatment period. d) Immunohistochemical assay of Hsp70 expression in tumor tissues of different treatment groups. e) Histological observation of the tumor tissues with H&E staining from different treated groups of mice. f) Weights of tumors harvested from different groups after treatment. Data were given as mean ± SD (*n* = 10), **P <0.05.

Finally, the antitumor efficacy was investigated using athymic nude mice bearing U87MG tumors at axilla sites. Mice in different groups were intravenously treated with HGN, HGN‐N, and HGN‐siHsp70, respectively, followed by photothermal therapy upon NIR laser irradiation. Tumor volumes, survival rates, and body weights of mice were monitored during the 15 d therapy (**Figure** **6**a–c). Certain extent of tumor volume reduction was found in mice treated with bare HGN and HGN conjugated with nonsense RNAs after photothermal therapy (HGN‐Laser and HGN‐N‐Laser group), but the survival rate of mice was not effectively improved. In addition, no significant difference in tumor inhibition was noted between the two groups, although more heat may be generated in the HGN‐N‐Laser group as evidenced in the above experiments. This was probably due to increased expression of Hsp70 in tumor tissues, which shielded cancer cells from damage of heat in this study (around 46 °C). In contrast, the HGN‐siHsp70‐Laser group showed the most pronounced delay of tumor growth compared with other groups, with more than 70% of mice surviving at day 15. The remarkable tumor inhibition efficacy in this group was mostly attributed to the downregulation of Hsp70 that sensitized tumor to hyperthermia, even at a moderate level around 46 °C. The in vivo knockdown of Hsp70 was then confirmed by immunohistochemical analysis (Figure 6d). The HGN‐Laser and HGN‐N‐Laser groups showed evidently increased Hsp70 expression compared with the saline control. The HGN‐siHsp70‐Laser group, however, did not display obvious change of Hsp70 level after photothermal therapy. Therefore, the nanoconstructs could effectively downregulate Hsp70 in vivo during photothermal therapy, which explained the excellent therapeutic efficacy of such a treatment strategy. The hematoxylin and eosin (H&E) staining of tumor tissues and weights of tumors harvested after treatment further confirmed the most potent therapeutic efficacy in HGN‐siHsp70‐Laser group (Figure 6e,f). Therefore, this photothermal platform could serve as a versatile and potent tool for NIR light activated gene silencing and improved photothermal therapy.

## 3 Conclusion

In this study, we have successfully developed a potent and versatile PTT platform based on hollow gold nanoshells functionalized with small interfering RNA against Hsp70. The nanoplatform was stable under biological environment and releases siRNAs with laser control. The cellular uptake study indicated that siRNA coating could substantially increase the intracellular accumulation of gold nanoshells, which may be associated with scavenger receptors and was considered as a contribution to enhanced photothermal efficacy. Effective downregulation of Hsp70 was achieved in light‐triggered cells but not observed in those without laser treatment. These results were finally translated into evidently improved tumor cell ablation of the siRNA‐nanoshells compared with the bare nanoshells or nonsense RNA‐nanoshells in vitro and in vivo.

Conclusively, these nanoconstructs have exhibited three unique characteristics that are synergistic to improve PTT efficacy. First, surface coating of oligonucleotide sequences can substantially increase the cellular uptake of the nanoparticles. Second, gold nanoshells can facilitate efficient siRNA delivery into cytoplasm with endosomal escape by thermalizing Au—S bond. Third, effective downregulation of Hsp70 in vitro and in vivo sensitizes cancer cells to heat. In addition, it is found that the uptake of siRNA coated gold nanoshells was mainly through scavenger receptors, which furthers the mechanistic research of siRNA delivery by gold nanoparticles and implies the potential of targeting scavenger receptors to enhance endocytosis.^37^ More importantly, as gold nanoshells have already been under clinical trials, this strategy, which features multiple functions in a quite simple system, offers easier quality control and inspires the translational application of such a strategy in cancer therapy.

## 4 Experimental Section

*Reagents*: Cobalt chloride hexahydrate, chloroauric acid (HAuCl_4_), trisodium citrate dehydrate, and sodium borohydride were purchased from Guoyao Reagent Corporation (Shanghai, China). Dithiothreitol (DTT) and glutathione (GSH) were purchased from Sigma‐Aldrich Inc. (USA). Sulfydryl polyethylene glycol, polyinosinic acid (Poly I), and methyl‐β‐cyclodextrin were purchased from Shanghai Sangon Biotech, Inc. Human malignant glioma cell line (U87MG), human breast cancer cell line (MDA‐MB‐231) were obtained from KeyGen Biotech. (Nanjing, China). Normal and athymic nude mice were purchased from SLAC Laboratory Animal Co. Ltd. (Shanghai, China). Bovine serum albumin and cell culture media were purchased from Sangon Biotech (Shanghai) Co., Ltd. The siRNAs against heat shock protein and nonsense sequences were purchased from Sangon Biological Engineering Technology & Co. Ltd. (Shanghai, China). The company also accomplished the modification of siRNAs and nonsense sequences with —SH and FAM.

Hsp70 siRNA: 5′‐CCAUUGAGGAGGUAGAUUAdTdT‐3′

Nonsense sequence: 5′‐GUACCAAUCCUACUGCCGAdTdT‐3′

*Equipment*: Morphological characterization of gold nanoshells was accomplished by transmission electron microscopic imaging on a Philips FEI Tecnai G2 20s‐TWIN (Netherlands) with an accelerating voltage of 200 kV. Particle size measurement was obtained on a Mastersizer 2000 laser particle size analyzer (Malvern, UK). Zetasizer Nano instrument (Malvern Instruments, Malvern, UK) was used to examine the zeta potential. The UV–vis and fluorescence spectra were obtained by a 754‐PC UV–vis spectrophotometer (JingHua Technological Instrument Corporation, China) and a spectrofluorophotometer (RF‐5301PC, Shimadzu, Japan), respectively. Cell imaging was performed using the laser confocal fluorescence microscope (FV1000, Olympus, Japan). The data of flow cytometry were obtained by FACS Calibur (Bioscience, America). The amount of gold in cells was determined by ICP‐AES (Optima 5300DV, Perkin Elmer, USA).

*Synthesis and Characterization of Hollow Gold Nanoshells Modified siRNAs and PEG*: HGNs were prepared according to the previously reported method with modification. In the first step, the sacrificing templates, cobalt nanoparticles were prepared with attention paid to air exclusion and cleanliness of glassware. Deionized water (100 mL) in a three‐necked bottle was added with sodium citrate (800 µL, 0.1 mol L^−1^) and cobalt chloride (200 µL, 0.4 mol L^−1^), and then the solution was bubbled with nitrogen for at least 40 min to deoxygenate. Afterward, the freshly prepared sodium borohydride solution (200 µL, 1 mol L^−1^) was added to the above mentioned solution with magnetic stirring. The pink solution of cobalt chloride turned to dark brown, which proved the formation of cobalt nanoparticles. The solution of cobalt nanoparticles was stirred for another 90 min to ensure the complete hydrolysis of sodium borohydride. The second step was to form gold nanoshells by adding gold salt to the solution of cobalt nanoparticles. Chloroauric acid could be easily reduced by sodium borohydride, so the complete hydrolysis of sodium borohydride was important. After inspecting the hydrolysis of sodium borohydride, chloroauric acid (875 µL, 0.024 mol L^−1^) was quickly added to the solution by a 1 mL syringe through the rubber plug inserted in the middle neck of the flask, with increased nitrogen flow and more rapid stirring. After chloroauric acid was added, nitrogen flow was stopped and the solution was open to air. The remaining cobalt nanoparticles were oxidized by oxygen in air, and the final products, hollow gold nanoshells were formed. The siRNAs and nonsense sequences were added to the HGN solution followed by 4 h stirring. SH—PEG (0.1 nmol L^−1^) was then added and incubated in the dark. Thereafter, sodium chloride solution was added at a final concentration (0.2 mol L^−1^) to stabilize the construct. The reaction solution was centrifuged at 14 000 rpm for 15 min to remove the unattached ligands. The supernatants were collected for quantification of loading amount. The final molar ratio of HGN:siRNA was 1:200. To prove siRNA and HGN were connected by Au—S bond, dithiothreitol (DTT) was added to the prepared HGN‐siHsp70 solution to a final concentration (0.1 mol L^−1^). After 2 h, the samples were centrifuged and the supernatants were collected for fluorescence analysis. Morphological characterization of the nanoparticles was performed on TEM (Philips FEI Tecnai G2 20s‐TWIN, Netherlands). Particle size and zeta potential were measured by Mastersizer 2000 laser particle size analyzer (Malvern, UK). The UV–vis absorption spectra were determined by 754‐PC UV–vis spectrophotometer (JingHua Technological Instrument Corporation, China).

*Determination of Nanoparticle Numbers in the Solution*: As the hollow gold nanoshells were spherical nanoparticles, the following two equations were used to determine the number of nanoparticles in solution

(1) $$U=\frac{2}{3}\pi \left[{\left(\frac{D_{1}}{\alpha }\right)}^{3}-{\left(\frac{D_{2}}{\alpha }\right)}^{3}\right]$$

(2) $$N=\frac{M}{U}$$

In the first step, the amount of gold atom in each hollow gold nanoshell was calculated according to Equation (1), where the average particle diameter and shell thickness were estimated through TEM images (particle diameter of about 50 nm and shell thickness about 7 nm). Then, the number of gold nanoshells in sample solution could be calculated following Equation (2). In these equations, α refers to unit cell edge of gold that has a value of 4.0786 Å. *D*_1_ refers to the diameter of the whole hollow gold nanoshell; *D*_2_ refers to the diameter of the hollow core; *M* refers to the gold atom number in solution determined by ICP‐AES; *N* refers to the number of gold nanoshells in sample solution.

*Laser Induced siRNA Release and Stability Test*: The prepared HGN‐siHsp70 solution (1 mL) was subjected to 660, 765, 808, and 980 nm laser with power density of 4 W cm^−2^ for 20, 40, 60, 80, 100, and 120 s. Then, the solutions were centrifuged at 14 000 rpm for 15 min and the supernatants were collected for quantification or spectra analysis. The release of siRNA triggered by 765 nm laser was further examined at 1, 2, 4, 6, and 8 W cm^−2^. For the stability test, GSH (15 × 10^−3^ m), DNase (0.4 mg L^−1^), RNase (0.02 mg L^−1^), and mice serum (0.5 mL) were, respectively, added into HGN‐siHsp70 solutions followed by fluorescence measurement of the supernatants. The stability of hollow gold nanoshells during laser irradiation was tested by subjecting HGN solutions to 765 nm laser irradiation at 4 W cm^−2^ for 2, 4, 6, and 8 min, respectively. The UV–vis spectrum at each time point was measured. The hydrodynamic diameters of the control HGN solution and the sample after 8 min laser irradiation were determined by DLS.

*Determination of the Photothermal Effect in Solution*: 765 nm laser with power density of 4 W cm^−2^ was used to induce the temperature change of the nanoshell solutions. Temperature change of the HGN solutions (100 µL) upon 765 nm laser irradiation was measured by a thermocouple which was inserted into the solution perpendicular to the light path. The temperatures were recorded in 10 min, with PBS solution as control.

*Cellular Uptake of the Nanoconstructs*: U87MG cells were seeded in confocal specific dish of 5 × 10^4^ cells per dish. Cells were treated with 0.5 mL of different constructs (HGN, HGN‐N sequence, and HGN‐siHsp70) of 2 × 10^−9^ m for 30, 60, 90, and 120 min, respectively. The mediums were removed at different time points, washed for three times with cold PBS, and fixed with 10% paraformaldehyde for 30 min. The cellular uptake of the nanoparticles was imaged under Olympus microscope system and CytoViva hyperspectral imaging system. For ICP‐AES analysis, U87MG and MDA‐MB‐231 cells were seeded in six‐plate well and incubated with different constructs (5 × 10^−9^ m), respectively. After 2 h incubation, cells were washed with cold PBS and enzymatically detached from the plates. Cells were then dissolved by aqua regia for ICP‐AES analysis. For the blocking studies, cells in 24‐well plates were pretreated with methyl‐β‐cyclodextrin (12.5 mg mL^−1^) or Poly I (50 µg mL^−1^) for 30 min. After PBS washing, solution of HGN‐siHsp70 (0.5 mL) was added for laser confocal imaging.

*Gene Silencing Assay by RT‐PCR and Western Blot*: U87MG cells were seeded in 24‐well plates at 5 × 10^4^ cells per well. Cells of seven groups were, respectively, treated with PBS (Group 1), HGN+6 min laser (Group 2), HGN‐N+6 min laser (Group 3), HGN‐siHsp70 (Group 4), HGN‐siHsp70+90 s laser (Group 5), HGN‐siHsp70+6 min laser (Group 6), and Lipo2000+siHsp70 (Group 7). The amount of siRNAs and nonsense RNAs delivered by gold nanoshells and Lipofectamine2000 was 5 × 10^−9^ m for 5000 cells. Group 1 with PBS only and Group 7 with siRNAs delivered by Lipofectamine2000 were used as negative and positive controls, respectively. 90 s laser was used to induce RNA release but not temperature increase that might influence cell viability. 6 min laser was used to exert photothermal effect and induce siRNA release at the same time. 24 h after incubation, all the groups except PBS control were exposed to 765 nm laser at 4 W cm^−2^. Then, 8 h after laser treatment, cells were harvested followed by mRNA and protein extraction. The levels of Hsp70 mRNA and protein were determined by real time PCR and western blot.

*Analysis of Cell Viability and Apoptosis*: U87MG cells were seeded and cultured in 96‐well plates to the density of 1 × 10^4^ cells per well. Cells were treated as the gene silencing assay indicated above for 48 h. Then, cells were washed with PBS and incubated with fresh medium containing MTT solution (20 µL, 5 mg mL^−1^). After 4h incubation, 150 µL dimethyl sulfoxide (DMSO) was added after removal of the medium. Absorbance was measured at wavelength of 570 nm using a microplate reader and cell viability was then calculated. For apoptosis assay of PTT, cells were plated at a density of 4 × 10^5^ cells per dish in confocal specific culture dish and then incubated for 36 h. The cells were then treated with Laser, HGN‐Laser, HGN‐N‐Laser, and HGN‐siHsp70‐Laser (RNA concentration of 5 × 10^−9^ m, 8 min irradiation of 4 W cm^−2^). After media removal and PBS washing, cells were then stained by annexin V‐fluorescein isothiocyanate (annexin V‐FITC) and propidium iodide (PI). Laser confocal microscopy and flow cytometry were, respectively, used to measure the apoptosis conditions according to the protocols.

*Conjugation of Near Infrared Dye to Gold Nanoshells*: The near infrared dye, ICG‐Der‐02 (*M*_W_: 995) was prepared in the laboratory. The —COOH group of ICG‐Der‐02 (240 mg) was activated by 1‐ethyl‐3‐[3‐dimethylaminopropyl]carbodiimide hydrochloride (EDC) and N‐hydroxysuccinimide (NHS) catalyst systems (molar ratio of carboxyl group:EDC:NHS = 1:1.5:1.5) in aqueous solution under continuous stirring for 2 h at room temperature. The constructs (HGN, HGN‐siHsp70) modified with NH_2_—PEG—SH was then added and the mixture was proceeded at room temperature for another 12 h and then centrifuged at 15 000 rpm for 10 min to remove the unreacted dye. The final products were named was HGN‐NIRdye and HGN‐siHsp70‐NIRdye.

*In Vivo Biodistribution and Photothermal Effect Assay*: All animal experiments were conducted according to the Animal Management Rules of the Ministry of Health of the People's Republic of China (document No. 55, 2001) and the guidelines for the Care and Use of Laboratory Animals of the China Pharmaceutical University. The mice model was established by injecting a suspension of about 5 × 10^6^ U87MG cells subcutaneously injected into the axillary fossa of each mouse. In vivo study began when the tumors reached 0.5 cm in diameter. The mice were randomly assigned into two groups. Tumor bearing mice in Group 1 and Group 2 were intravenously injected with HGN‐siHsp70‐dye (1.5 × 10^−9^ m kg^−1^) and HGN‐dye (1.5 × 10^−9^ m kg^−1^) and then subjected to near infrared fluorescence imaging system at 15 min, 2 h, 6 h, 12 h, and 24 h. For the assay of in vivo photothermal effects, 200 µL of HGN and HGN‐siHsp70 (1.5 × 10^−9^ m kg^−1^) were i.v. injected to tumor bearing mice for three times every 12 h. Then, at 36 h, tumor sites of the mice were irradiated with 765 nm laser for 8 min. The thermal camera (Fluke Co. Ltd., USA) was used to record the temperature changes of tumor area within 8 min.

*In Vivo Photothermal Therapy*: For in vivo photothermal therapy, nude mice bearing U87MG tumors were randomly divided into four groups with ten mice in each: (1). Saline control; (2). i.v. injection of HGN (1.5 × 10^−9^ m kg^−1^) with 8 min laser irradiation; (3). i.v. injection of HGN‐N (1.5 × 10^−9^ m kg^−1^) with 8 min laser irradiation at 4 W cm^−2^. (4). i.v. injection of HGN‐N (1.5 × 10^−9^ m kg^−1^) with 8 min laser irradiation. The administration of all the nanoconstructs was 200 µL per 12 h for three times. Tumor sizes were measured using a digital caliper. Tumor volume was calculated as width^2^ × length/2. After treatment, the tissue sections were stained with H&E and observed under the brightfield microscopy (Olympus, Japan). For immunohistochemistry assay, sample tumors were collected 24 h after treatment for subsequent Hsp70 immunohistochemistry, according to the manufacturer's instructions (KeyGen Biotech, Nanjing, China).

## Supporting information

As a service to our authors and readers, this journal provides supporting information supplied by the authors. Such materials are peer reviewed and may be re‐organized for online delivery, but are not copy‐edited or typeset. Technical support issues arising from supporting information (other than missing files) should be addressed to the authors.

Supplementary
