# Supplementary material for: Laser‐Triggered Small Interfering RNA Releasing Gold Nanoshells against Heat Shock Protein for Sensitized Photothermal Therapy
Source: Adv Sci (Weinh). 2016 Oct 19;4(2):1600327. doi: 10.1002/advs.201600327 (PMC5323853; doi:10.1002/advs.201600327)
Supplement: Supplementary file 1 — Supplementary [file ADVS-4-na-s001.pdf]

Copyright WILEY-VCH Verlag GmbH & Co. KGaA, 69469 Weinheim, Germany, 2016.

ADVANCED SCIENCE
Open Access

Supporting Information

for *Adv. Sci.*, DOI: 10.1002/advs.201600327

Laser-Triggered Small Interfering RNA Releasing Gold Nanoshells against Heat Shock Protein for Sensitized Photothermal Therapy

*Zhaohui Wang, Siwen Li, Min Zhang, Yi Ma, Yuxi Liu, Weidong Gao, Jiaqi Zhang, and Yueqing Gu**

Copyright WILEY-VCH Verlag GmbH & Co. KGaA, 69469 Weinheim, Germany, 2013.

Supporting Information

Laser Triggered Small Interfering RNA Releasing Gold Nanoshells against Heat Shock Protein for Sensitized Photothermal Therapy

*Zhaohui Wang[#], Siwen Li[#], Min Zhang, Yi Ma, Yuxi Liu, Weidong Gao, Jiaqi Zhang and Yueqing Gu**

State Key Laboratory of Natural Medicines, Jiangsu Key Laboratory of Drug Screening,

Department of Biomedicine Engineering, School of Engineering,

China Pharmaceutical University,

No. 24 Tongjia Lane, Gulou District, Nanjing 210009 (China)

E-mail: guyueqingsubmission@hotmail.com

[#]These authors contributed equally to this work

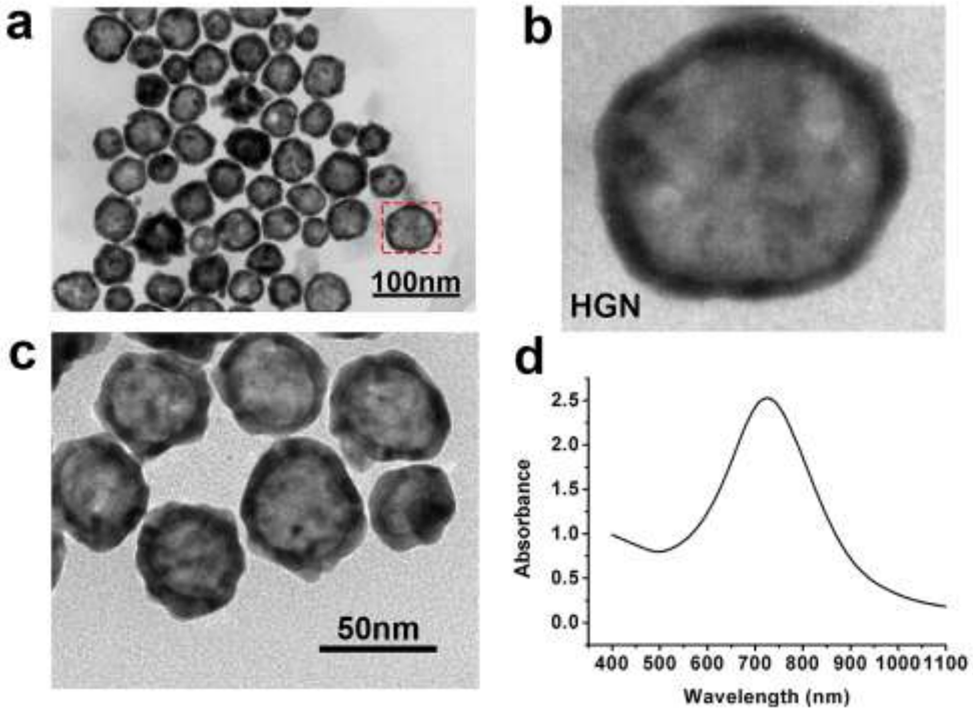

Figure S1. Characterization of hollow gold nanoshells (HGN). a), b) and c) TEM images of HGN in different magnifications. Red box in a) indicates the single particle in b); d) UV-vis absorption of HGN.

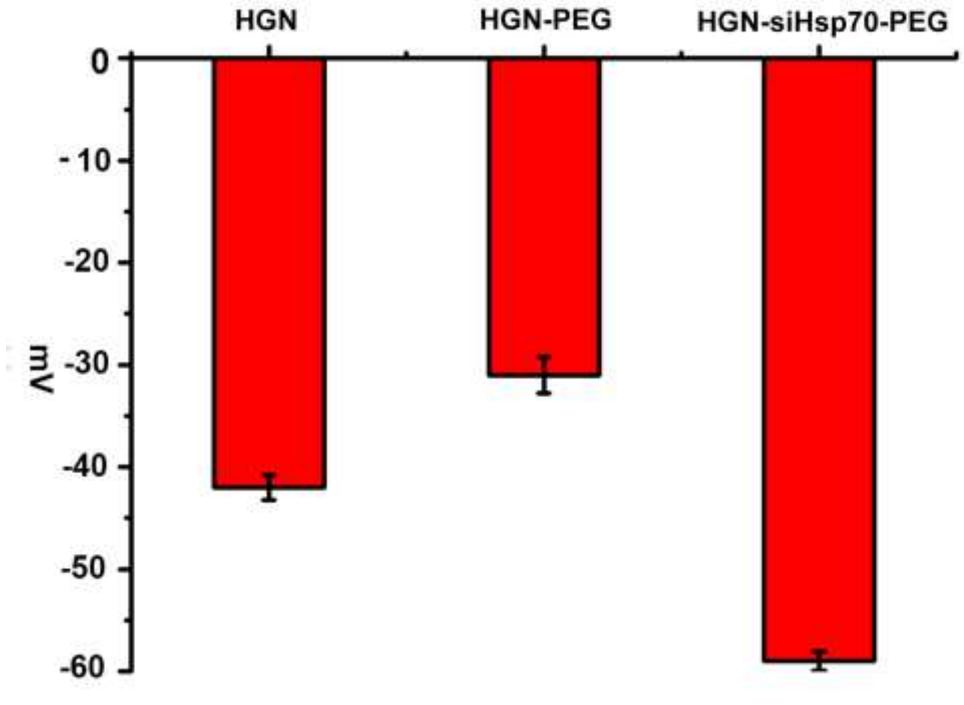

Figure S2. Zeta potentials of HGN, HGN-PEG and HGN-siHsp70-PEG.

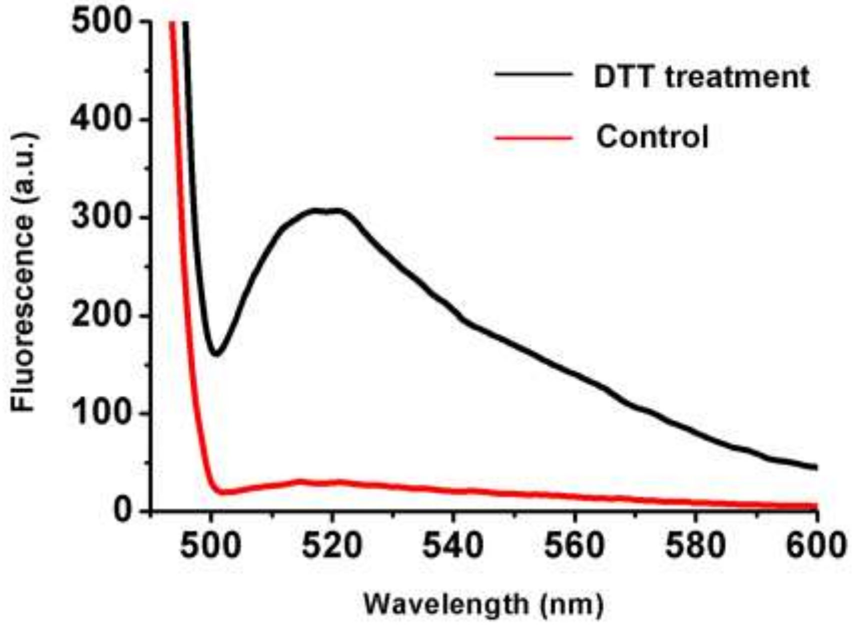

Figure S3. Release of siRNA from HGN triggered by DTT treatment. a) Fluorescence spectra of HGN-siHsp70 treated with DTT (black curve) and the control solution (red curve).

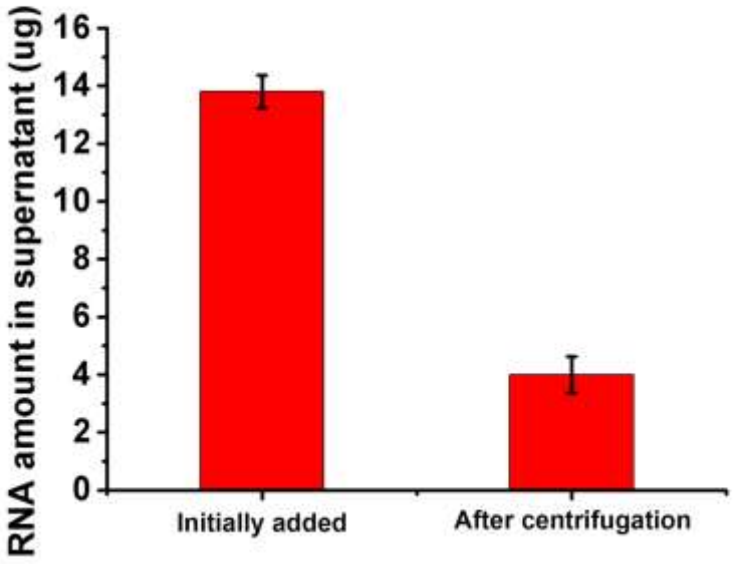

Figure S4. Quantification of siRNAs coated on the surface of HGNs. The initially added siRNA in the solution before reaction, and the amount of siRNA left in the supernatant were quantified respectively.

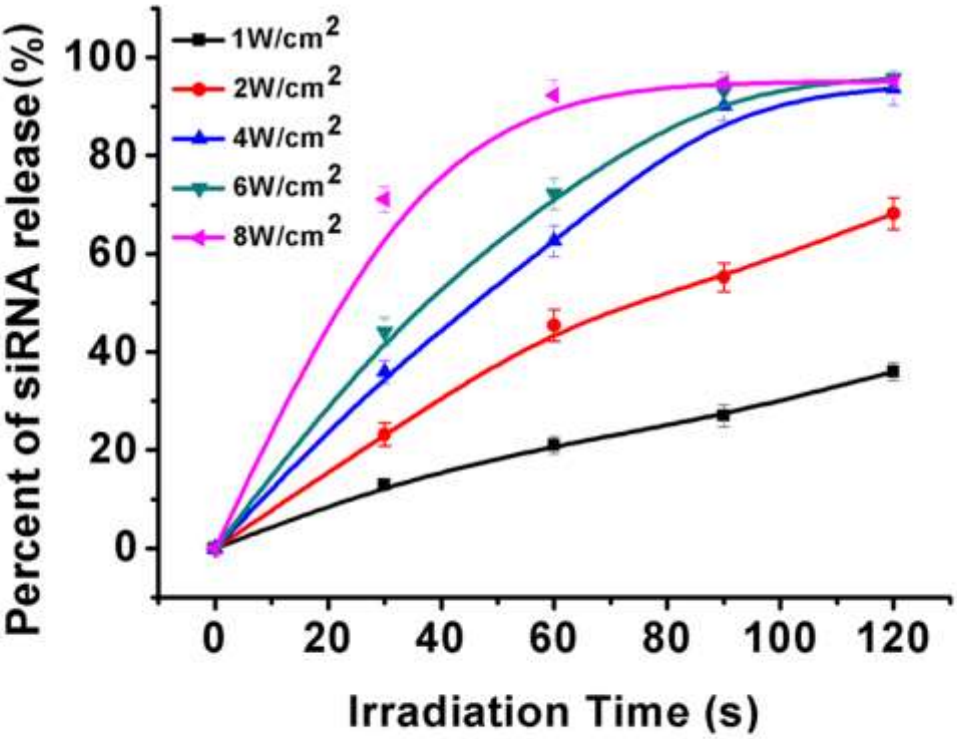

Figure S5. Percent of siRNA release from HGN-siRNA under 765nm laser of different power densities.

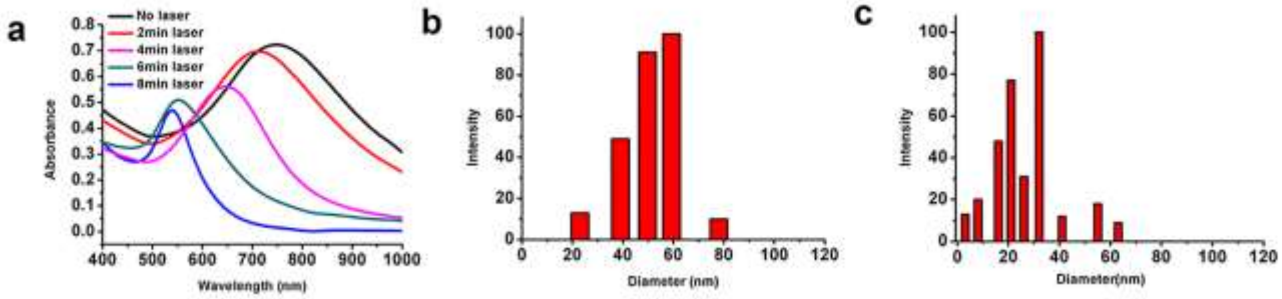

Figure S6. Photothermal stability of HGN during 765nm laser irradiation. a) UV-vis spectrum of HGN subjected to laser irradiation for different periods of time; b) hydrodynamic diameter of HGN before laser irradiation; c) hydrodynamic diameter of HGN after laser irradiation.

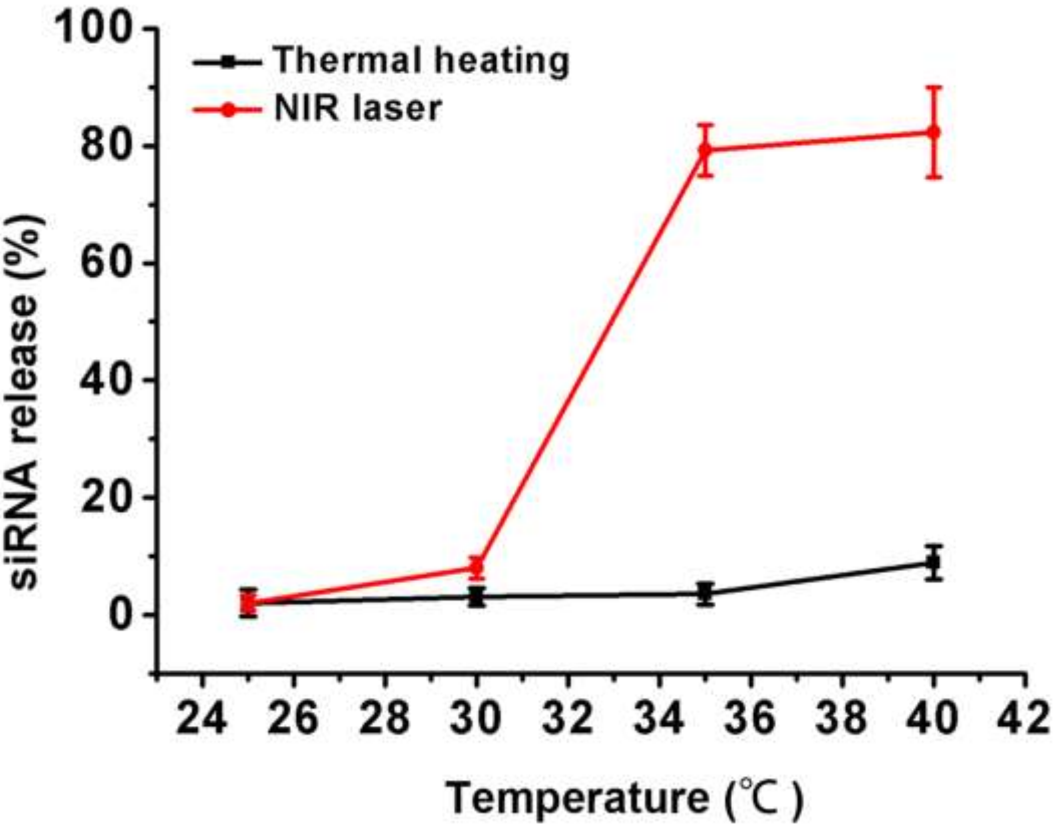

Figure S7. Different release profiles of siRNA with direct thermal heating and NIR laser irradiation.

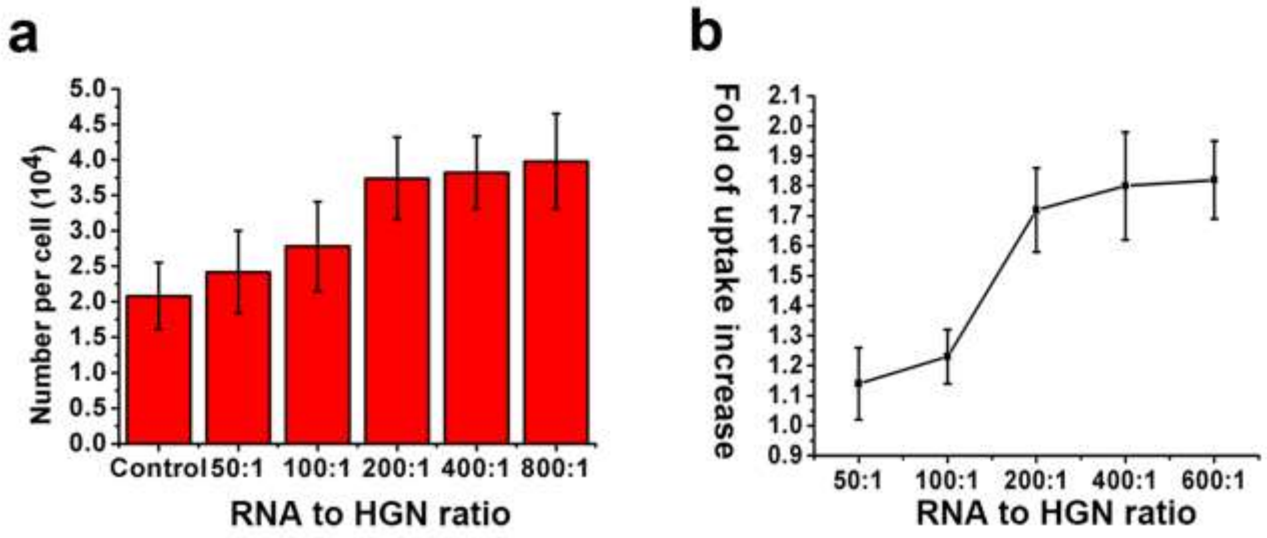

Figure S8. ICP-AES quantification of the cellular uptake of HGN with different numbers of RNA coating. a) Number of HGN per cell; b) Fold of cellular uptake increase at different RNA to HGN ratios.
